# Resection of a Giant Middle Mediastinal Tumor Using a Transaortopulmonary Arterial Approach

**DOI:** 10.1016/j.atssr.2025.04.006

**Published:** 2025-05-08

**Authors:** Kaito Yano, Tsutomu Ito, Keisuke Asakura, Yuriko Tanabe, Yorihiko Matsumoto, Kaoru Kaseda, Yutaka Kurebayashi, Yoshitake Yamada, Tomoyuki Hishida, Hideyuki Shimizu

**Affiliations:** 1 Department of Thoracic Surgery, Keio University School of Medicine, Tokyo, Japan; 2 Department of Cardiovascular Surgery, Keio University School of Medicine, Tokyo, Japan; 3 Department of Pathology, Keio University School of Medicine, Tokyo, Japan; 4 Department of Radiology, Keio University School of Medicine, Tokyo, Japan

## Abstract

Surgical approaches for giant middle mediastinal tumors are challenging owing to the surrounding great vessels and vital organs. Here, we report the resection of a giant middle mediastinal tumor with feeding vessels from the coronary artery causing exertional dyspnea. Surgical resection involved a clamshell incision and temporary transection of the ascending aorta and right pulmonary artery under cardiopulmonary bypass to achieve optimal visualization. The tumor, diagnosed as unicentric Castleman disease, was safely excised with good postoperative outcomes. This method—the transaortopulmonary arterial approach—offers a favorable surgical tool for giant mediastinal tumors.

Middle mediastinal tumors include lymphoproliferative diseases, neurogenic tumors, and sarcomas, among others. Surgical resection is indicated when malignancy is suspected or when the tumor compresses surrounding organs.^1^ Giant middle mediastinal tumors are surrounded by the ascending aorta (AA), pulmonary artery (PA), heart, bronchi, and vertebrae, making their resection challenging.^2^ Furthermore, owing to the potential for invasion, adhesion, and blood supply from surrounding organs and vessels, ensuring clear visualization of the entire tumor is critically important for safe resection. Herein, we report the resection of a giant middle mediastinal tumor with feeding vessels from the coronary artery, involving temporal transection of the AA and right PA under cardiopulmonary bypass (CPB).

A 44-year-old man, who presented with abnormal shadow on a chest roentgenogram and exertional dyspnea, was revealed to have an 11.5- × 11.0- × 8.1-cm middle mediastinal tumor on chest computed tomography (CT) (Figure 1A). Positron emission tomography/CT showed moderate fluorodeoxyglucose uptake (Figure 1B). A specimen from a right thoracoscopy-assisted biopsy revealed fibrosis and atypical cells. Three-dimensional CT showed feeding vessels in the left coronary artery (Figure 1C).

**Figure 1:**
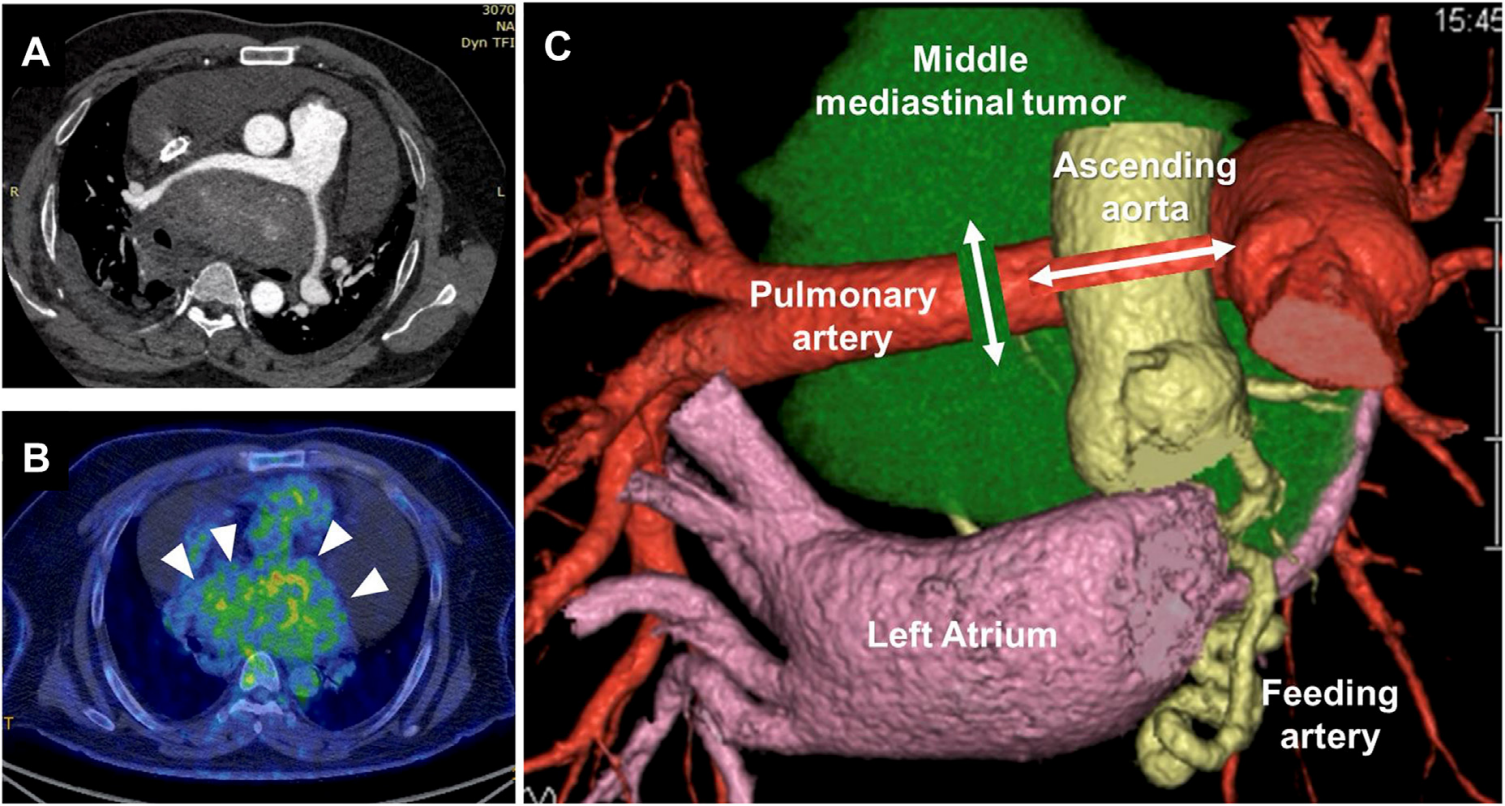
(A) Computed tomography (CT) shows a giant middle mediastinal tumor. (B) Positron emission tomography-CT shows moderate fluorodeoxyglucose uptake (arrowheads). (C) Three-dimensional CT shows feeders from the left coronary artery and transecting sites of the ascending aorta and right pulmonary artery.

Surgical resection was planned to address the compression of the surrounding organs and exertional dyspnea. Securing a proper contralateral view to manage the feeding arteries would have been difficult using a lateral approach. Therefore, an anterior approach was chosen in which the AA and right PA were temporarily transected to obtain optimal visualization of the tumor (Figure 1C). This surgery was approved by the Keio University School of Medicine Institutional Ethics Committee (May 9, 2024, approval number: 93).

A clamshell incision was made. Rapid pathologic diagnosis by excising a part of the tumor revealed atypical cells forming vascular-like structures compatible with both a malignant and benign lesion; thus, we decided to proceed with tumor resection. CPB was established with dual venous drainage from the superior vena cava and inferior vena cava and arterial perfusion through the femoral artery.

After cardiac arrest induction, the AA was transected, followed by transection of the right PA. The tumor was dissected from the posterior surfaces of the AA, right PA, bronchi, and heart. The tumor was excised by ligating the feeding vessels. The transected right PA was reconstructed using sutures, followed by AA reconstruction. Cardiac activity resumed after anastomosis completion.

The operation time was 8 hours and 53 minutes, the CPB time was 3 hours and 52 minutes, the aortic cross-clamp time was 2 hours and 15 minutes, and the total blood loss was 808 mL. The operative video is available as a supplemental file (Video).

The patient experienced no significant postoperative complications and was discharged on postoperative day 16. The postoperative 3-dimensional CT confirmed good patency of the reconstructed sites of the AA and right PA (Figure 2).

**Figure 2:**
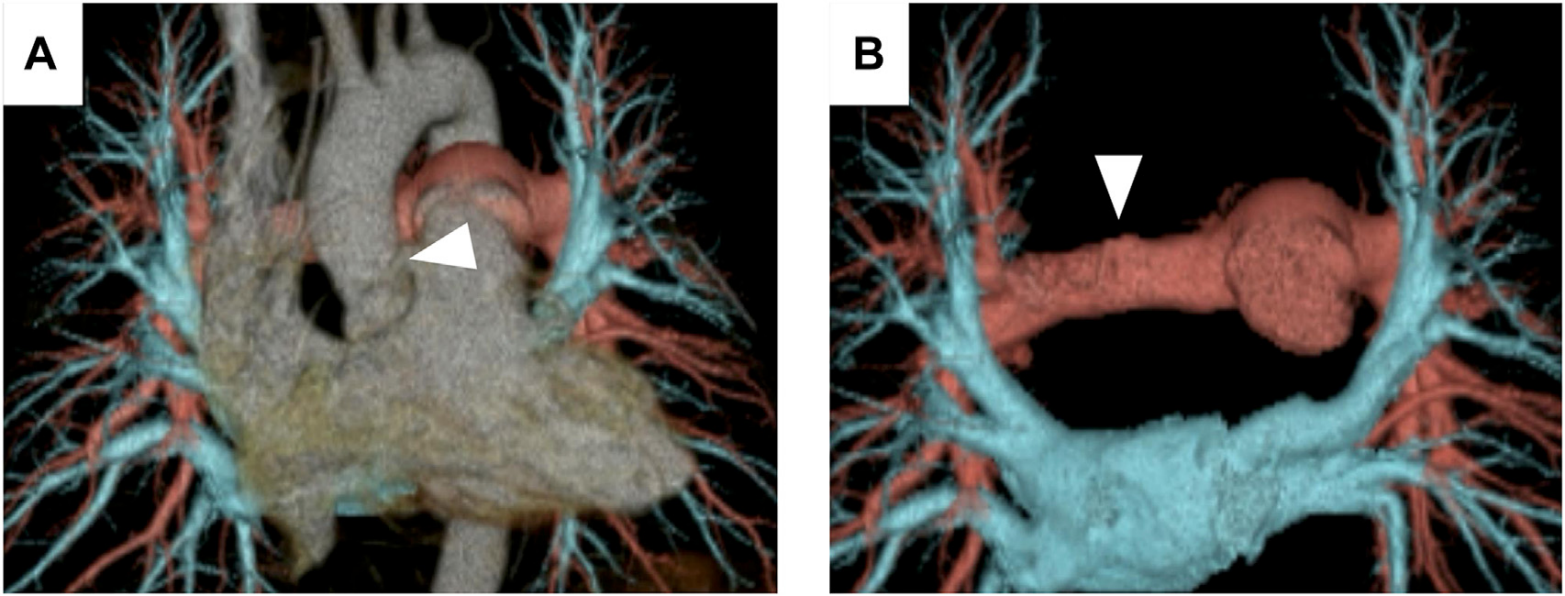
Three-dimensional computed tomography images obtained on postoperative day 5. (A) Reconstructed ascending aorta shows good patency (arrowhead). (B) Reconstructed right pulmonary artery shows good patency (arrowhead).

In histopathologic examinations, the tumor was diagnosed as unicentric Castleman disease (Figure 3). The tumor was well encapsulated from the surrounding fibrous tissue, and the surgical margin was negative. The patient is being monitored by the Hematology Department, without any treatment or recurrence at 7 months after surgery.

**Figure 3:**
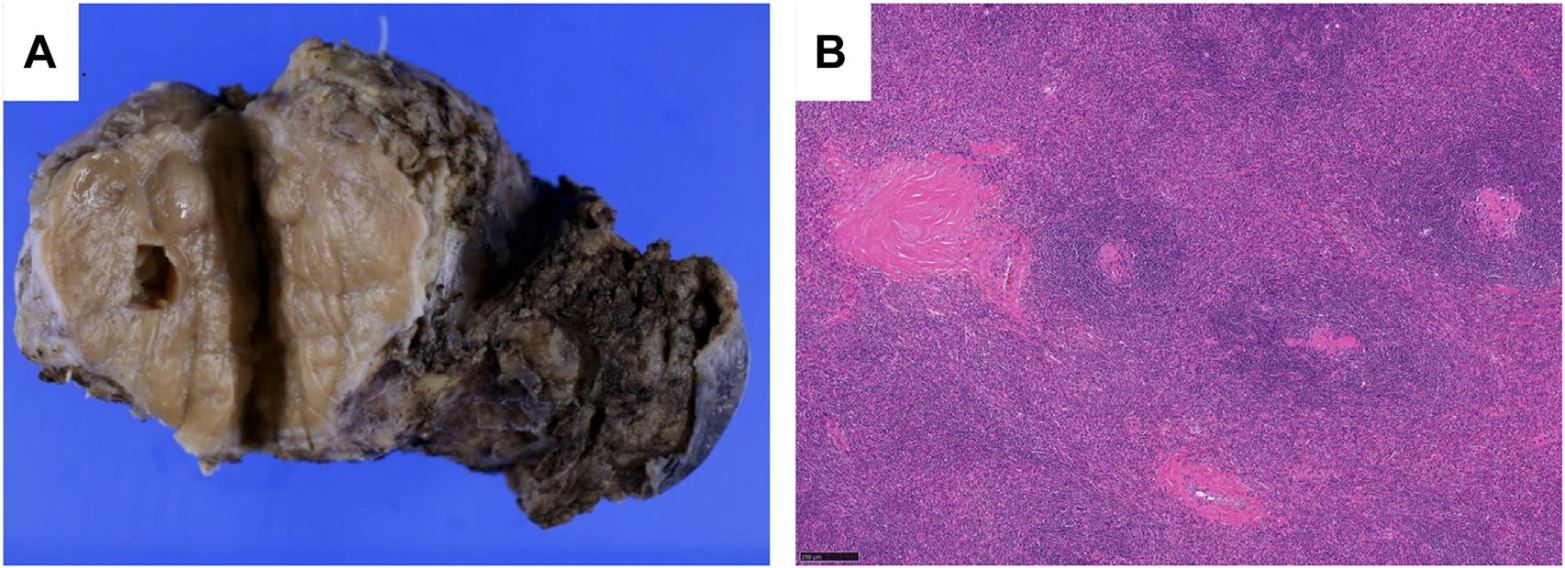
Histopathologic findings of the mediastinal tumor. (A) Macroscopic appearance. (B) Histopathologic examination revealed follicular structures with hyalinized blood vessels, demonstrating the features of hyaline vascular-type Castleman disease (hematoxylin and eosin, original magnification ×40).

## Comment

The standard treatment for unicentric Castleman disease is surgical resection, and the prognosis is considered favorable after complete resection.^3^ On the other hand, for unresectable unicentric Castleman disease with symptomatic compression, pharmacologic therapy is indicated.^4^ In this case, although a definitive diagnosis was not established preoperatively, malignancy was suspected based on the surgical biopsy specimen, and the patient also presented with exertional dyspnea. Therefore, despite the anticipated challenges, the decision to proceed with surgical treatment was made.

Surgical approaches for giant middle mediastinal tumors are challenging owing to the surrounding great vessels and vital organs.^2^ Xu and colleagues^5^ reported the resection of an 11.5-cm middle mediastinal tumor through a right thoracotomy, with 2400 mL of blood loss from the feeding vessels. Collaud and colleagues^6^ reported the resection of an 11-cm middle mediastinal tumor using a right thoracotomy; however, the tumor was located behind the lung (almost posterior mediastinal tumor) and accessible from the right thorax. Velders and colleagues^7^ reported the transpericardial resection of an 8.4-cm middle mediastinal tumor with the feeders from the aortic arch through a median sternotomy while avoiding the AA and right PA. Ishibashi and colleagues^8^ approached an 8.4-cm middle mediastinal tumor through a median sternotomy, considering the possibility of left PA invasion. The tumor was divided into 2 parts across the aorta and resected.^8^ From these reports, it is evident that in the resection of giant mediastinal tumors, careful consideration of the surgical approach to secure a clear surgical field and ensure proper management of the feeding vessels is a critical challenge.

In this case, the tumor was an 11.5-cm middle mediastinal mass with feeding vessels derived from the coronary arteries. Considering the possibility of adhesion, a lateral thoracotomy approach was anticipated to present significant difficulties in managing the feeding vessels and in dissecting the tumor from the contralateral bronchi, PA, and pulmonary vein. Furthermore, because the tumor was located directly behind the AA and right PA, it was anticipated that a traditional anterior approach would make it difficult to secure an adequate surgical field. Therefore, a surgical method—the transaortopulmonary arterial approach—was devised, involving a clamshell incision and temporary transection of the AA and right PA under CPB to access the tumor. This approach allowed for the establishment of an adequate surgical field encompassing the entire giant tumor, enabling the safe dissection of the tumor from the AA, PA, pulmonary vein, and bronchi, as well as the secure management of the feeding vessels.

Acknowledging the inherent invasiveness of this surgical procedure is crucial. Further, a preoperative assessment is essential to ensure adequate cardiopulmonary function and confirm the absence of calcification or other pathologic conditions in the ascending aorta that may lead to anastomotic complications and embolization. Continued accumulation of cases and further evaluation of this technique are required.

In conclusion, the transaortopulmonary arterial approach can be useful for the resection of giant middle mediastinal tumors.
